# Technique for Early Reliability Prediction of Software Components Using Behaviour Models

**DOI:** 10.1371/journal.pone.0163346

**Published:** 2016-09-26

**Authors:** Awad Ali, Dayang N. A. Jawawi, Mohd Adham Isa, Muhammad Imran Babar

**Affiliations:** 1 Department of Information Technology, Faculty of Computer Science, University of Kassala, Kassala, Sudan; 2 Department of Software Engineering, Faculty of Computing, Universiti Teknologi Malaysia, UTM, 81310 Skudai, Johor, Malaysia; 3 Department of Computer Sciences, Army Public College of Management & Sciences Rawalpindi, Pakistan; Southwest University, CHINA

## Abstract

Behaviour models are the most commonly used input for predicting the reliability of a software system at the early design stage. A component behaviour model reveals the structure and behaviour of the component during the execution of system-level functionalities. There are various challenges related to component reliability prediction at the early design stage based on behaviour models. For example, most of the current reliability techniques do not provide fine-grained sequential behaviour models of individual components and fail to consider the loop entry and exit points in the reliability computation. Moreover, some of the current techniques do not tackle the problem of operational data unavailability and the lack of analysis results that can be valuable for software architects at the early design stage. This paper proposes a reliability prediction technique that, pragmatically, synthesizes system behaviour in the form of a state machine, given a set of scenarios and corresponding constraints as input. The state machine is utilized as a base for generating the component-relevant operational data. The state machine is also used as a source for identifying the nodes and edges of a component probabilistic dependency graph (CPDG). Based on the CPDG, a stack-based algorithm is used to compute the reliability. The proposed technique is evaluated by a comparison with existing techniques and the application of sensitivity analysis to a robotic wheelchair system as a case study. The results indicate that the proposed technique is more relevant at the early design stage compared to existing works, and can provide a more realistic and meaningful prediction.

## Introduction

Observation of the trends in a range of fields indicates a variety of computer software applications. Computer software can be found embedded in many devices and equipment, such as hand phones, automobiles and aircraft. In addition, software is increasingly used to support critical business applications and industrial processes. Most of these fields depend on software for their basic functioning. Software failure can lead to critical events and fatal consequences in safety-critical applications as well as in business applications. In order to meet customer expectations and needs, the software must have high reliability. The increasing demands of software functionalities are leading to various issues including the scalability and degree of concurrency of the software system. Customer satisfaction is also a serious challenge; thus, software reliability engineering must live up to the needs of today’s complex software systems and their specific challenges [1].

The reliability approach is formalized to explain the failure behaviour within a system. Software reliability is defined as the probability that the software system will perform a required function correctly (failure-free) in a stated environment for a specified period of time. Due to the heterogeneity of the execution environment and the development methodology of current software systems, a failure broadly can mean that the software system is unable to deliver the expected service and is not capable of resuming its service to the state prior to being interrupted. Several kinds of failures are possible during service execution, such as faults in the implementation of the software components, hardware failure and network failure. Hardware failure is due to an unreliable hardware resource, and network failure occurs because the message is lost or there is a problem in inter-component communication [2,3]. Predicting software reliability at an early design stage enables the software’s designer to identify and improve any weak design spots. This is more cost-effective than fixing consequent errors at later implementation phases. Therefore, the reliability technique must be able to work at the early design stage, and particularly during the architectural design phase.

Based on the lifecycle of the reliability measurement, the reliability measures taken early while building and later during the testing or post-deployment [4,5,6]. The data used in these measurements respectively are appraisal data, testing data, and real world data. The purpose of the early measurement is to discover the design spots in order to rework, while later measurements are used for the release decision or to certify the components or the whole system. There is no difference in the capability and the property of the reliability approaches that can be used for the two types of the later measurements, because the input data and the purposes are similar. For instance, the later approaches mainly focus on the prediction accuracy while do not pay more attention to the methods of data elicitation and behaviour modeling. Meanwhile, the early approaches concentrate on how to tackle the problem of lack of operational data before the coding stage and the precise modeling of the system behaviour [2,7].

A software development team is a cohesive coalition of individuals working together towards a common goal [8]. The structure of the development team may consist of sub-teams such as design, implementation and deployment team. The members of these sub-teams are requirements analysts, architects, coders, component engineers, testers and so on. The number of the members often depends on the project size and the company policy. For instance, in a small project, the number of the members could be small. Therefore, a team member may have to play a number of roles, either at the same time or in frequent alternation. The early measurement of the reliability is part of the design process and stage, therefore, the reliability analysis is conducted as part of the design process [9]. In turn, the later measurements are part of the testing process and stage, hence, the analysis is implemented as part of the testing. The reliability approach in this research is used early as part of the design activities.

Based on behaviour models, several techniques can be used to evaluate the reliability of software at an early stage and identify the reliability-critical elements of the architecture. However, the existing techniques suffer from a number of drawbacks that limit their applicability and accuracy.

First, according to [2,10] several existing techniques use imprecise, coarse-grained, sequential models of system architecture as the base for early reliability prediction. Imprecise and coarse-grained terms are referring to the use of Markov chains directly as a modeling notation to create the architectural models in the form of a state machine. In this sense, system or component states are represented and interpreted by the state machine, with neither any intermediate notation that reveals the concurrent nature of the system architecture (such as UML, SysML) nor an explicit mechanism that defines how the state machine was constructed.

Second, most of the existing techniques [7,10,11,12] model the influence of the loop entry and exit points on the control and data flow throughout the component behaviour model while neglecting that during reliability computation, because they use Markov model to compute the reliability which assumes the state transition probabilities are history-independent. For example, if a specific set of components’ operations invokes more than another set, because it represents a sequence in a loop, and if these operations have a higher failure rate than the other operations, then computing the reliability without considering the number of invocations will produce an inaccurate prediction. Therefore, the use of technique that is able to keep the previous invocations related values may produce more accurate results. This paper’s work attempts to remedy the shortcomings of the early reliability prediction techniques by proposing a technique for predicting component reliability based on fine-grained sequential models of system architecture synthesized from scenario specifications. This technique is intended to be complementary to the existing approaches of system-level reliability prediction. The values obtained from the proposed technique can be used in existing (or future) system-level reliability approaches which require the reliability values of the newly designed components. We argue that dealing with the important challenges in component reliability prediction at the early design stage stems from the precise derivation of an architectural model that is able to reveal the components’ structural and behavioural perspectives, tackle the unavailability of operational data and consider the loop entry and exit points of the behaviour models in the reliability computation.

The paper is structured as follows. We first highlight the research gap in early reliability prediction and then discuss the related works in Section 2. Section 3 defines the proposed technique’s elements and construction steps and illustrates the applicability of the proposed technique using an illustrative example. In Section 4, we evaluate and illustrate the applicability of the proposed technique using a real world case study. The last section concludes the paper and provides an outline of future works.

## Related Works

During the last decade, many techniques have been proposed to predict software reliability in the early design stage depending on behavioural models; these techniques address different problems and challenges. However, individual component reliability is an integral issue that should be considered in predicting the reliability of a software system at the early design stage [13,14]. Except for certain works [3,10,11,15], which we discuss in this section, most of the current approaches [6,16,17,18,19,20,21,22,23,24] predict the reliability of a system based on the reliability of its components, without going into sufficient detail about the internal behaviours of the components with respect to down-to-up prediction. It appears that these works assume the availability of the operational data related to individual components. The operational data can be used to determine component reliability accurately without considering a component’s internal structure and behaviour; however, sometimes such information is not available at the early design stage (e.g.in the case of brand new components).

The work by [3,10,11,15] can be seen as a precursor of our technique because these scholars provide explicitly early reliability techniques for predicting individual components using behavioural models. These approaches consider the effects of a component’s internal structure and behaviour in terms of its reliability. The works by [3] and [15] employed parameter dependencies and the components’ environment or the deployment environment to predict component reliability. These works demonstrate the enhancement of reliability prediction via documentation of the component services’ external and internal behaviour in a structured control flow manner. The work by [3] is one of the few approaches that compare the predicted values and the actual measured values for evaluating the accuracy of the approach. The behaviour of the component in [15] is documented by the component’s developers as call-propagation over a component service and input parameter values. The documentation is conducted via stochastic regular expressions with the probability of failure for each internal action. This mechanism makes the capture of transition dependencies mathematically tractable, even in the case of complex components. However, this approach does not specify how the input values can be obtained [13,25].

On the other hand, the work by [11] proposes a modeling approach that can be used for developing a representative operational profile that tackles the lack of knowledge about the new component’s operational data at the early design stage. The profile is built based on the domain expert and operational data obtained from similar functional component(s). In the same way, the work by [7] addresses the problem of individual component reliability prediction at the early design stage and the operational data unavailability. Furthermore, the author modifies the operational profile devolved in [11] by using multiple information sources that can be available at the early design stage, such as the requirements specification document and a simulation technique in order to achieve more accuracy. The adoption of the work in [7] as part of the system-level reliability approaches [12,25] demonstrates the need for the prediction of individual component reliability in order to predict the whole system reliability. The component reliability techniques in [11] and [7] mapped the component’s states to a first-order discrete-time Markov chain (DTMC) in order to compute the reliability. However, the first-order DTMC does not explicitly reflect the effects of architectural features such as loops and conditional branching in the component reliability prediction [12]. Moreover, none of these techniques use a fine-grained method that utilizes the explicit requirements specification as the main source at the early design stage to synthesize the behaviour models. The scenario-based method of Rodrigues et al. [10] is perhaps closest in spirit to our own technique. In that work, the behaviour model of the component is synthesized from the requirements specification. The requirements are provided in scenarios using message sequence charts (MSCs). Then, the states of the behaviour model are mapped to the DTMC to compute the reliability. However, that work did not consider the influence of loop entry and exit points in the computation, due to the use of the DTMC.

## Proposed Technique for Component Reliability Prediction

The reliability of a component is predicted based on the component’s architectural design and the operational data relevant to this design. The component architectural design is modeled or constructed in the form of a state machine. This state machine can be derived from the code using induction algorithms or from the requirements specification using behaviour synthesis algorithms. This paper’s work is intended for the early design stage before the coding stage; therefore, the proposed technique is built through the behaviour synthesis. Synthesizing a behaviour model or deriving a state machine from requirements specification is the starting point for the proposed technique.

**Fig 1:**
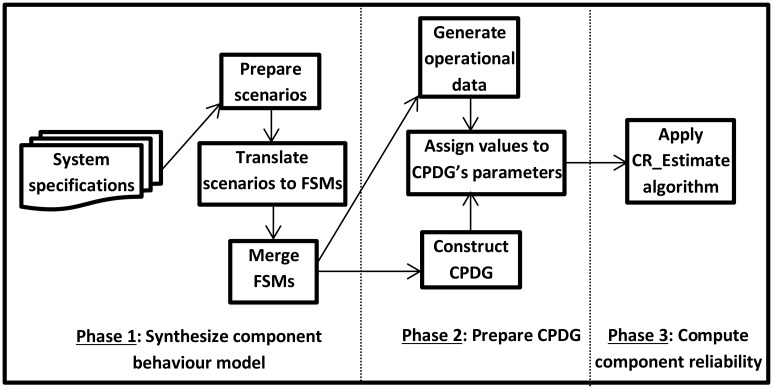
Phases of software component reliability prediction.

For ease of exposition, the proposed technique is depicted as a three-phase process as shown in Fig 1. Broadly, the requirements specification is the main source utilized by the technique to synthesize the component behaviour model. Finite state machines (FSMs) are the basic elements that used in the behaviour synthesis. The behaviour model can be used for two purposes: as a simulation of the component behaviour and as a source for obtaining and identifying the elements of a probabilistic dependency graph. The simulation provides an execution log for the component, and the log serves as the runtime observation data required as input to generate operational data for the component. The operational data are necessary to determine the values of the dependency graph’s parameters. Finally, the constructed graph (which is a component probabilistic dependency graph (CPDG)) is used as input to a tree transversal algorithm which works to compute the component reliability.

A dependency graph is selected to represent the component structure and behaviour for two reasons. First, it facilitates the capture and modeling of an individual component’s behaviour (including the loop entry and exit points) and includes the consideration of that information in the reliability computation. This aspect is overlooked in most current component reliability techniques. Second, it is typical to use a specific computation algorithm, namely, the tree transversal algorithm, to allow for a tractable solution.

### 3. Phase 1: Synthesizing the component behaviour model

The process of synthesizing the component behaviour from scenario specifications as a popular requirements elicitation tool involves three activities: preparing scenarios, translating the component instances in each scenario to FSMs, and merging the FSMs of each component into one state machine model such as the labelled transition system (LTS). In order to define how the behaviour models can be synthesized, this section briefly reviews our previous research work [26], which is relevant to the synthesis of behaviour models from requirements specification; noting that the technique proposed in this paper is not dependent only on our previous work. Any behaviour model that is obtained through one of the existing behaviour synthesis methods or even from a component’s code as a result of a reverse engineering process can be used.

#### 3.1.1 Preparing scenarios

**Fig 2:**
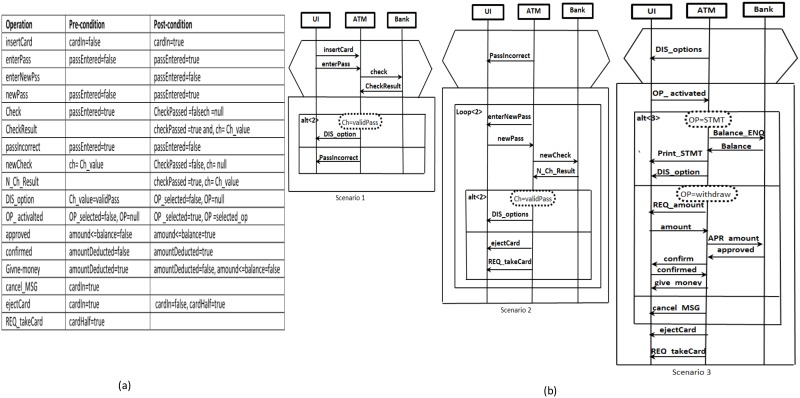
System specifications: (a) ATM system constraints, (b) ATM system scenarios using s-TSs.

Briefly, the system scenarios in this paper’s work were written using a scenario language called the scalable triggered scenario (s-TSs) language. Triggered scenario languages provide syntactic constructs for describing the conditional or causal relations between sequences of actions. Scenarios in a language like live sequence charts, are described in conditional form (called universal form) and existential. In triggered language, scenarios are described in universal form with existential semantics. This type of modeling provides a good fit with use cases which is the primary form of requirements elicitation. An example of an existential scenario is the automatic teller machine (ATM) scenario which describes a statement like “If the user inserts a valid card into the ATM, and then enters the correct password, she/he shall be able to request cash and have it dispensed by the ATM”. This statement is also conditional in the sense that requesting and obtaining cash is expected to be possible if the user has inserted a valid card and input the correct password. An example of a universal statement is: “If the user inserts a valid card into the ATM, and then enters an invalid password, then she/he must receive the password incorrect message”. The s-TSs facilitates the writing of statements like “If the user inserts a valid card into the ATM, and then enters a valid password, then she/he must able to see the ATM options, otherwise she/he must receive the password incorrect message”. The last statement in a universal form, but more concise and compact (two universal statements combined together). s-TSs use constructs such as implied triggers and branching messages to compact the statements. Fig 2 shows the specifications of the ATM system, with Fig 2(a) depicting the system constraints which are elicited as domain knowledge, and Fig 2(b) illustrating the ATM scenarios using s-TSs.

**Fig 3:**
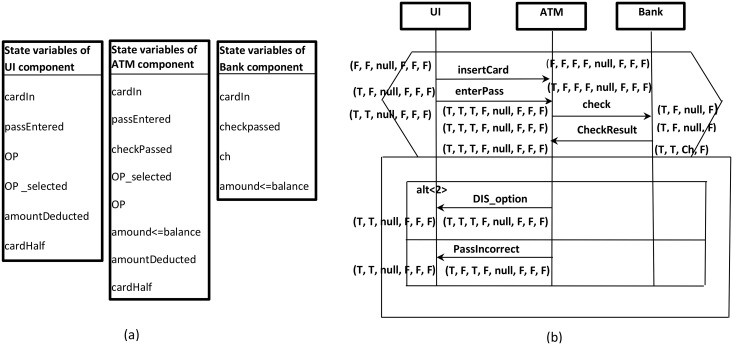
Scenario preparation: (a) Elicited state variables of each component in the ATM example, (b) Scenario1 of Fig 2 after annotation based on the state variables and propagation.

The s-TSs enhance the current triggered scenario languages [27,28] by adding constructs that enable the writing of scenarios in a compact and concise manner in order to enhance the scalability of scenario modeling. At the early design stage when complete information about the behaviour of a system is not available, there is no option other than to leverage the system constraints and their state variables as basic information sources to enrich system scenarios which are already documented using s-TSs as mentioned previously. The constraints and their state variables (held in a system state vector) are elicited as domain knowledge related to the early design’s specifications. In order to prepare scenarios based on these information sources, in Step 1 we elicit a component’s state variables from the system state vector. The component’s state variables are used to define the constraints relevant to the component’s incoming and outgoing messages. In Step 2, the values of the component’s state variables which appear in the constraints table (Fig 2(a)) are used to annotate scenarios as pre- and post-conditions associated with each incoming and outgoing message of the component instance. Each component instance is annotated independently, depending on its own state variables list. The reason for this independence is that the goal is only to construct the behaviour model of the component (not the behaviour of the system that represented through the scenario). The values of some state variables may be marked as missing due to not having specifications. Thus, these missing values in the annotated scenarios need to be propagated in Step 3 of this phase using a propagation technique similar to the work by [29] and [30]. Fig 3(b) shows one of the scenarios in Fig 2 after implementing the scenario preparation steps.

#### 3.1.2 Translating the component instances within the scenario to a set of FSMs

**Fig 4:**
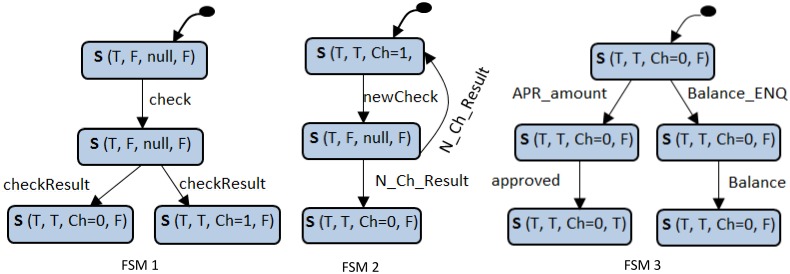
FSMs of the “Bank” component in the ATM system (obtained from the three scenarios in Fig 2).

Once the scenarios are prepared (annotated and propagated), we are ready to synthesize a behaviour model for each component in the system. The strategy starts by generating a number of FSMs for the component (one FSM from each scenario). Thus, each FSM represents the behaviour of the component corresponding to a specific scenario from the set of system scenarios. These FSMs will later be merged (in Phase3) to produce a complete behaviour model of the component. In order to convert each component instance within a scenario to FSM, pre-post conditions values and operations (incoming and outgoing messages) of this component instance will be translated to states and transitions, respectively. Fig 4 shows the three FSMs of the “Bank” component obtained from the three ATM system scenarios shown previously in Fig 2**.**

#### 3.1.3 Merging the set of component FSMs into one state machine model

**Fig 5:**
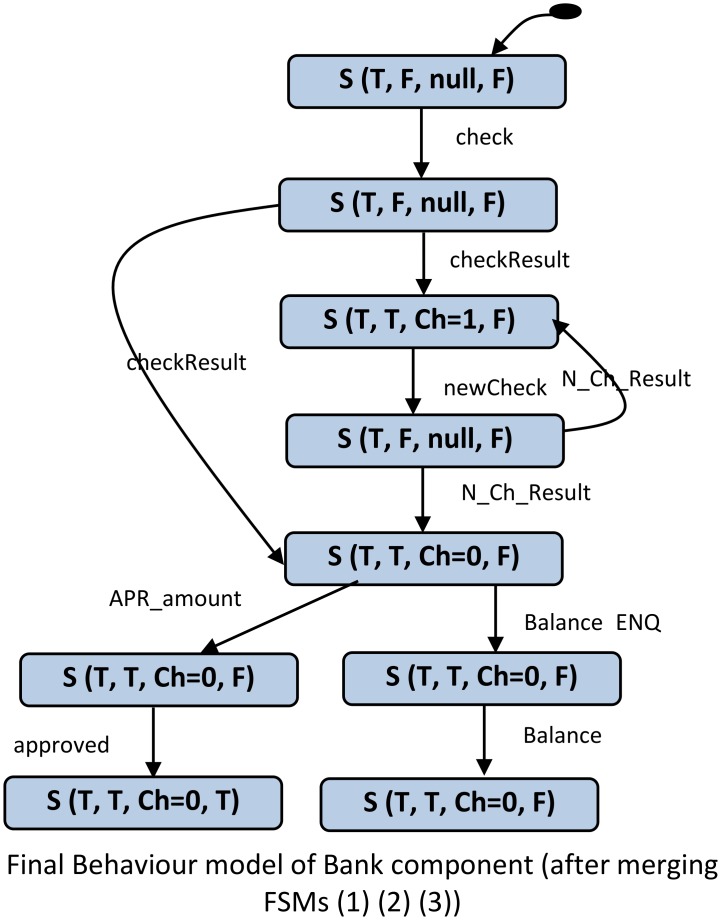
State machine of “Bank” component (as a result of merging the FSMs shown in Fig 4).

In the final activity in behaviour model construction, we merge the different FSMs of the component by identifying identical terminal and starting states. Two different FSMs will be merged if and only if the terminal state of one is similar to the starting state of the other. The merging transition will be created from a terminal to a start (the transition from a start to a terminal is not allowed). The similarity between the states is determined based on the state vector values of the states. The final output of this phase is the LTS which represents the behaviour of the component. Fig 5 shows the LTS as a result of merging the component FSMs of the “Bank” component shown above in Fig 4.

### Phase 2: Preparing a component probabilistic dependency graph

A CPDG is a directed graph that reveals the component’s structure and behaviour, which determine the component’s reliability. The use of a probabilistic graph is a classical method in software engineering applications. Baah et al. [31] propose a probabilistic graphical model that works with algorithm to analyze program behaviour. This model is used for a program’s fault comprehension and localization. In early reliability prediction there are a number of approaches that use a probabilistic graph. Yacoub et al. [18] proposed a dependency graph with a scenario-based algorithm as a technique to analyze the reliability of a component-based software system. However, the nodes in Yacoub et al.’s graph representing states of multiple components while CPDG states are belonging to one component, because the purpose in this research is to predict component reliability while in [13] the goal is the whole system reliability.

Preparing the CPDG involves two activities: constructing the CPDG, and generating the operational data. In the construction activity, all the elements of the CPDG are defined based on the basic notation and definitions of the CPDG and the synthesized behaviour model of the targeted component. The synthesis of the behaviour model was already described in relation to the previous phase. The next subsection defines the notations and parameters of the CPDG. Then, the operational data generation activity which provides the data used to assign values to all the CPDG parameters is described.

#### 3.2.1 Constructing the CPDG

Briefly, the CPDG construction requires the identification of its basic notation and definitions. In graph theory, a directed graph G is defined as a set of pairs, G = (N, E), where:

N represents a set of nodes

E represents a set of edges

For CPDG the formal definition is:

G = (N, E) where:

N is a finite set of nodes representing the component’s states

N = {*S*, Entry, Exit} where:

S is defined by the tuple <*S*_*i*_, *RS*_*i*_> where:

*S*_*i*_ is a unique identification of the component’s states

*RS*_*i*_ is the reliability of a state i (it is a probability that indicates that the component will pass the current state correctly (fault free)

Entry is a virtual state pointing to the first state of the component’s execution (it has no input transition and it reliability is 1)

Exit is a virtual state pointing to the termination of the component’s execution (it has no outgoing transition and it reliability is 1)

E is a finite set of edges representing the transitions between the component’s states

E = {T}.

T is defined by *PT*_*ij*_ or *PT*_*iExit*_ where:

*PT*_*ij*_ is the probability of transition from state i to state j, which is the probability that the next state will be executed after the current state (the sum of the outgoing transition probabilities from each state to all the other states, including implicitly the failure transition, should be 1)

*PT*_*iExit*_ is the probability of transition from state i to exit state.

**Fig 6:**
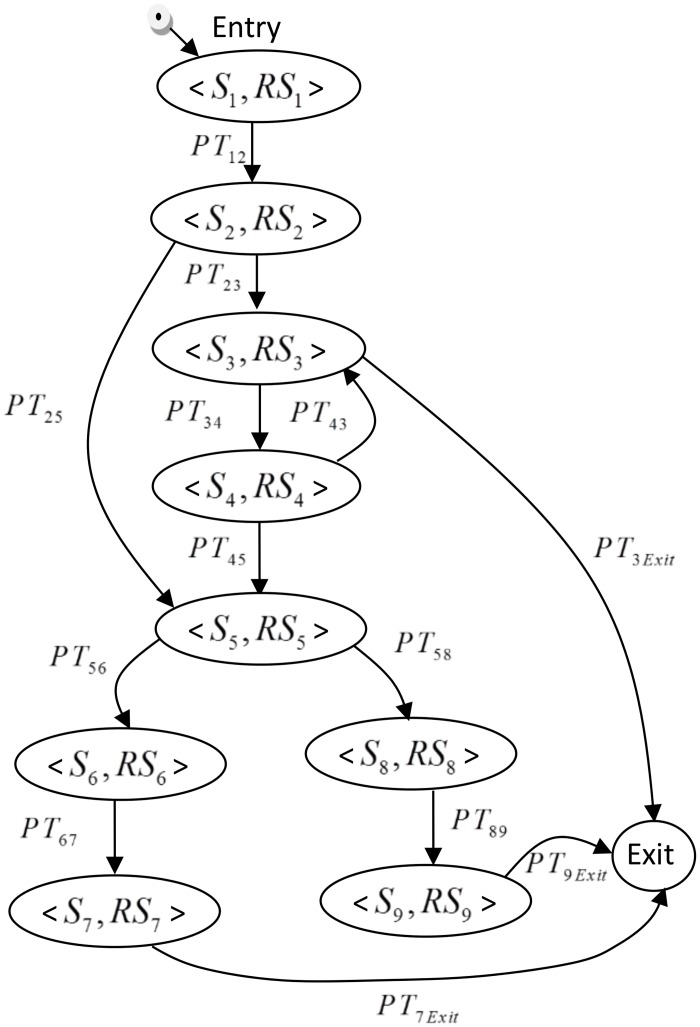
A CPDG constructed from the“Bank” component behaviour model.

Fig 6 shows an example of how a CPDG can be constructed based on the states and transitions of the behaviour model of a component. Fig 6 depicts the CPDG of the “Bank” component which is constructed using the behaviour model of this component. The nodes in the CPDG are directly inherited from the states of the behaviour model, whereby all the states in the behaviour model become nodes in the CPDG. Moreover, “super” nodes Entry and Exit are added to represent the initiation and termination of the execution.

#### 3.2.2 Generating the operational data

The operational data describe the behaviour of the component quantitatively. The data identify an ordered set of operations that the software component performs along with their associated probabilities. At the early stages of software development, the operational data on a given component may not be available, particularly in the case of newly designed components, and a design time reliability prediction technique must take this uncertainty into consideration. To generate operational data in this paper’s work, the concept of the representative operational profile that has been used in the literature [7,12], relying on a hidden Markov model (HMM) and a Baum–Welch algorithm[32], is adopted. The HMM is defined by four elements’ states S = {S_1_,S_2_,S_3_,…,S_n_}, a transition matrix A = {a_ij_}that represents the transition probabilities from state S_i_ to state S_j_, observations O = {O_1_, O_2_, O_3_, …, O_m_}, and an observation probability matrix E = {e_ik_} that represents the probability of observing event O_k_ in state S_i_. In this paper’s work, the component behaviour model that was synthesized in the previous phase will be mapped to define the HMM states S and transition matrix A = {a_ij_}. The observations O and the observation probability matrix E = {e_ik_} are identified based on the data gathered from similar function components, domain knowledge, and analysis of the component architectural model using the technique proposed by [33].

Baum–Welch is an iterative optimization technique used with HMM to approximate the best transition and observation probabilities. It is defined as an expectation–maximization algorithm that, given the number of states S, number of observations O, and a set of training data A = {a_ij_}and E = {e_ik_},gives the best values for the transition and observation probability matrices A and E. In brief, the data obtained from the Baum–Welch algorithm represent the operational data of the component based on the training data, which represent the component’s behaviour based on its architectural design. However, all these details are relevant to operational profile modeling, which is beyond this paper’s scope. The operational data are utilized directly to predict all the CPDG parameters; for instance, if the state S_i_ fails 5 times each 100 execution, it means the reliability of state i is (RS_i_ = 0.95). Similarly, the operational data give the frequencies of the transitions among the states which translate the transition probabilities PT_ij_ into the CPDG.

### Phase 3: Computing the component reliability

After constructing the CPDG and defining its related parameters, an algorithm to estimate the component reliability (CR_Estimate) is developed. The algorithm estimates or computes the component reliability based on the CPDG branches and their relevant parameters. In the CPDG, each path represents consecutive states and transitions. The algorithm traverses the CPDG thereby computing the paths’ reliabilities. The computation is based on Eq 3, which is derived from Eq 1. Eq 1 has been widely used by path-based reliability approaches [20,34,35,36] at the system level, while in this technique it is adopted at the component level. This adoption is similar to most of the state-based component reliability prediction techniques [7,11,12], which reuse a system-level formula at the component level. The CR_Estimate algorithm takes the CPDG and the components’ maximum expected iteration number as inputs (as the component operates for a long time). Its outputs are the components’ reliabilities with the iterations from zero to the maximum expected iteration number. As with the depth first searching algorithm, CR_Estimate traverses all the CPDG paths from Entry to Exit. Each path is iterated until the number of iterations equals the maximum number of expected iterations. The algorithm at each cycle of the computation refers to the number of the iteration and this determines the termination; therefore, in CR_Estimate, infinite loops that lead to deadlock are not allowed.

By adopting the formula in [35] the path reliability can be defined as:

$$R_{P_{k}}=\prod_{i=1}^{n}RS_{i}^{\text{Pr}({\text{v}}_{i})}$$ (1)

where:

$R_{p_{k}}$ is the reliability of the path number *k* where *k* = 1, 2, …, K.

*K* is the total number of paths.

*n* is the number of states in the path.

Pr(v_i_) is the probability of visiting each state i belonging to the path from the initial state. From the CPDG definitions, the probability of transition to the first state is 1; then Pr(v_i_) can be rewritten as:

$$\begin{array}{l} \text{Pr}(v_{i})=1.\text{P}{\text{T}}_{1,2}.\text{P}{\text{T}}_{2,3}.\ldots .\text{P}{\text{T}}_{i-2,i-1}.\text{P}{\text{T}}_{i-1,i}\\ =\text{P}{\text{T}}_{1,2}.\text{P}{\text{T}}_{2,3}.\ldots .\text{P}{\text{T}}_{i-2,i-1}.\text{P}{\text{T}}_{i-1,i}\\ =\prod_{i=2}^{i}\text{P}{\text{T}}_{i-1,i} \end{array}$$ (2)

$$∴R_{P_{k}}=R_{1}\times \prod_{i=2}^{n}RS_{i}^{\prod_{i=2}^{i}\text{P}{\text{T}}_{i-1,i}}$$ (3)

$$R_{c}=\frac{\sum_{k=1}^{K}R_{P_{k}}}{K}$$ (4)

**Algorithm 1**, beginning from the start node, computes the reliability of all the CPDG branches. As shown in Lines 13 and 14, the branch reliability is computed based on Eq 3. At the end of each branch, the reliability value of that branch is stored in an *Rtemp* variable(Line 8).Using the value of the *Rtemp*, the component reliability is then computed using Eq 4 (Line 23). Eq 4 is a common way to compute path reliability in most path-based reliability approaches [20,36,37].

**Algorithm 1** Component reliability estimation algorithm: CR_Estimate

1. **function** *computRc(Graph CPDG*, maximum expected iteration max_it)

2. ***Initialization***: pthTemp = 1,transTemp = 1, it_no = 0; Rtemp = 0;

3. **s = Stack.Create;**

4. s.push (*S*_*I*_, *RS*_*I*_, it_no, transTemp, pthTemp);

5.  **While** Stack ≠∅ **do**

6.   **s.pop**(*S*_*i*_, *RS*_*i*_,it_no, transTemp, pthTemp);

7.    **if** *S*_*i*_ = = Exit {Exit node}

8.     Rtemp+ = PthTemp;

9.     k++, it_no = 0;

10.    **else**

11     **for** it_no = 0 **to** max_it **do**

12.      **for all** *S*_*j*_, *RS*_*j*_ ∈ *S*_*i*_ successors **do**

13.       transTemp* = *PT*_i,j_;

14.       pthTemp* = power(*RS*_*j*_,transTemp);

15.       s.push(*S*_*j*_, *RS*_*j*_,it_no + = 1, pthTemp);

16.       **If** it_no = = max_it

17.        *S*_*i*_ = Exit;

18.       **end if**

19.      **end for**

20.     **end for**

21.    **end if**

22.   **end While**

23. *R*_*c*_ **=** Rtemp/k

24. **return** *R*_*c*_

25. **end function**

## Evaluation

This section presents the evaluation of the proposed technique in terms of applicability checking, sensitivity analysis and comparison evaluation. The applicability checking is directed to reveal whether the prediction of component reliability that is obtained based on the behaviour models synthesized from requirements specification is both possible and meaningful or not and furthermore to generalize the proposed technique. The results are demonstrated in the context of a real world case study. The sensitivity analysis is designed to show that the proposed technique may respond meaningfully to changes in its parameters, which in turn indicates to the correctness of the technique. The sensitivity analysis is also used to recognize the critical states in a component whose modification has a greater impact on improving the component reliability. From this perspective the sensitivity analysis can be shown as a decision support tool for evaluating various design alternatives. Finally, the comparative analysis was conducted to investigate the improvement yielded by the proposed technique with respect to the problems of existing techniques that have been discussed in the introduction of this paper.

### Case Study

In the evaluation we used a software component named the *avoid-component* which is part of the controlling system of a robotic wheelchair system [38]. The wheelchair software is a component-based system that has been developed by our research group to support research in embedded real-time (ERT) software engineering and rehabilitation robotics. The robotic wheelchair provides mobility for people with a disability and elderly people who are unable to operate the classical wheelchair system. The behaviour of the robot while in motion is highly constrained by the characteristics of reliability attributes and safety criteria. The robotic wheelchair consists of a motor power platform that is complete with a detector and certain movers, which depend on the suitability and usability to achieve wheelchair functionality. The most common detectors and movers, such as the infrared detector, sonar, laser, fibre optics and others, are used in the robotic wheelchair system to detect an obstacle and determine the distance. The power driving system is one of the important factors in a robotic wheelchair because the main purpose is to facilitate wheelchair consumer movement, along with other advantages such as driving automatically and avoiding obstacles.

In order to avoid unnecessary complexity, this research focuses mainly on activities that are related to the scenario of obstacle avoidance from the point of view of an avoid-component of a robotic wheelchair. In this scenario, the avoid-component receives a *detectObstacle* signal; this obstacle maybe on the left side or the right side. Depending on the position of the obstacle, the system has to activate an *obstacleLeft* or *obstacleRight* variable, which is located in a component called *Subsumption*. As soon as the variable is activated, it has to set a global variable named *avoidActive*, and then it has to wait 2 mc for a direction change before returning back to the *detectObstacle* state to repeat all these activities again.

### Synthesis of the Behaviour Model of avoid-component

**Fig 7:**
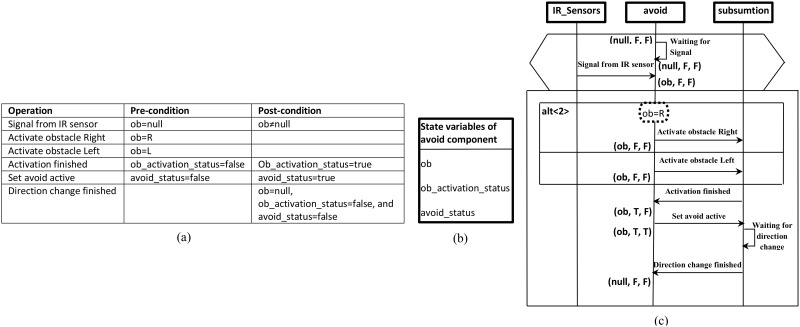
Part of wheelchair system specifications: (a) System constraints, (b) State vector of avoid-component (elicited based on (a) and the basic scenario of obstacle avoidance), (c) Prepared (annotated and propagated) scenario of obstacle avoidance to synthesize the behaviour model of the avoid-component.

**Fig 8:**
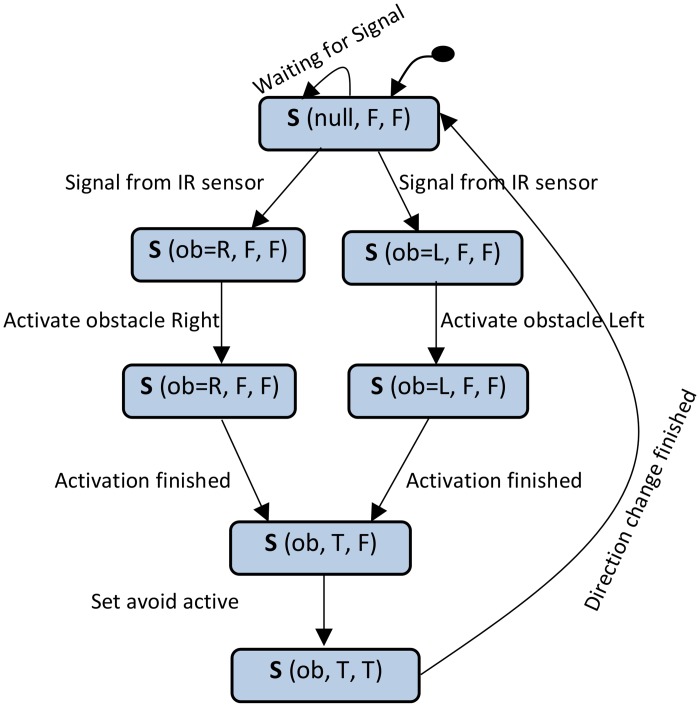
Behaviour model of the avoid-component.

Fig 7(a) shows the wheelchair system constraints as part of the requirements specification. Based on these constraints and the scenarios of the system, the state variables of the avoid-component (shown in Fig 7(b)) were elicited. Using system constraints and the state variables of the avoid-component, the scenario of obstacle avoidance shown in Fig 7(c) is prepared (annotated and propagated). By applying the steps defined previously and based on the prepared scenario of obstacle avoidance, the behaviour model of the avoid-component is constructed. This behaviour model is shown in Fig 8.

As an illustration, assume that the failure rate values in the behaviour model are related to the operations that appear in the scenario of obstacle avoidance (Fig 7(c)) for which the values are shown in Table 1. Similar to the work by [7,11], as described previously, these values were inferred from analogous components with similar operations and input obtained from a domain expert (the wheelchair developer). The failure rates and the behaviour model are required in the next phase to prepare the CPDG.

**Table 1:** Failure rate values of operations in the obstacle avoidance scenario.

Operation	Probability of failure
Signal from IR sensor	0.01
Activate obstacle Right	0.005
Activate obstacle Left	0.002
Activation finished	0.004
Set avoid active	0.004
Direction change finished	0.002

### Preparation of the CPDG of avoid-component

**Fig 9:**
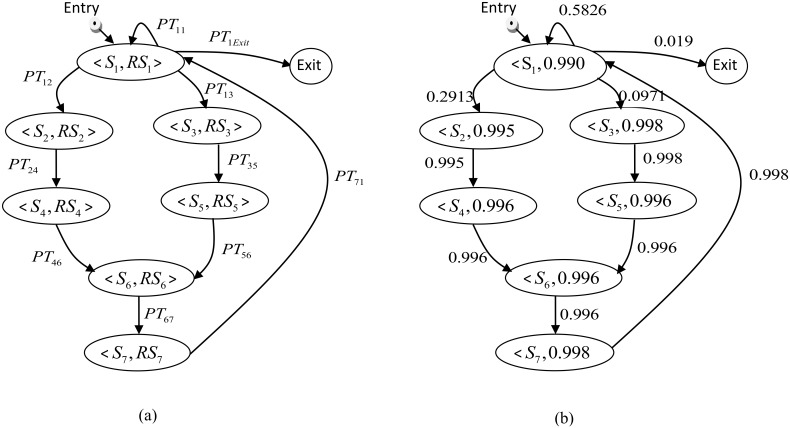
The CPDG of the avoid-component: (a) before and (b) after assigning the values of state reliablities and transtion probablities.

To prepare the CPDG of the avoid-component, two steps are needed: constructing the CPDG, and generating the operational data relevant to the component. The data are used to assign the values of the transition probabilities of the CPDG. The CPDG is constructed through mapping each state and transition in the behaviour model to the node and edge in the CPDG. The super nodes Entry and Exit are added to represent the instantiation and termination of execution. Fig 9(a) shows the constructed CPDG of the avoid-component.

Based on the states of the behaviour model and the failure rates shown above in Table 1, the operational data relevant to the avoid-component were obtained. As described previously, the data were generated through construction of a HMM and execution of the Baum–Welch algorithm to train the HMM. To build the HMM, sets of state S = {S1, S2, S3, …, Sn} and observations O = {O1, O2, O3, …, Om}are needed. Therefore, each state in the behaviour model is mapped to a state in S, and each transition is mapped to an observation in O. Similar to the work by [7,11,12], the domain knowledge and similar function components (e.g. the values in Table 1) are used to obtain the basic information that describes the behavioural transitions. This information is used as a basis to initialize the values of the HMM. For example, to determine the probability of receiving a signal from the IR sensor, the probability of failure relevant to this operation which is obtained from similar components is used. However, to determine the detail about whether the signal is received from the left or right IR sensor, domain knowledge is used. For instance, assume a domain expert mentioned that the signal comes from the left sensor most of the time. Therefore, receiving the signal from the left IR sensor can take higher probability than the right. After initializing the values of the HMM using this type of information and executing the Baum–Welch algorithm, the final transition probabilities required in the CPDG are obtained. Fig 9(b) shows the constructed CPDG of the avoid-component after assigning the transition probabilities.

### Computation of the Reliablity through Application of the CR_Estimate Algorithm

We implemented the CR_Estimate algorithm defined in Section 3.6 and applied it to the CPDG of the avoid-component (Fig 9). Our objective was to analyze the relationship between the execution cycle and the component reliability by solving the steady state probability of not being in any failure state (as the component operates for a long time). Moreover, we aimed to analyze the sensitivity of the component reliability to the states’ reliabilities. We also investigated how different usage scenarios affected the application reliability.

**Fig 10:**
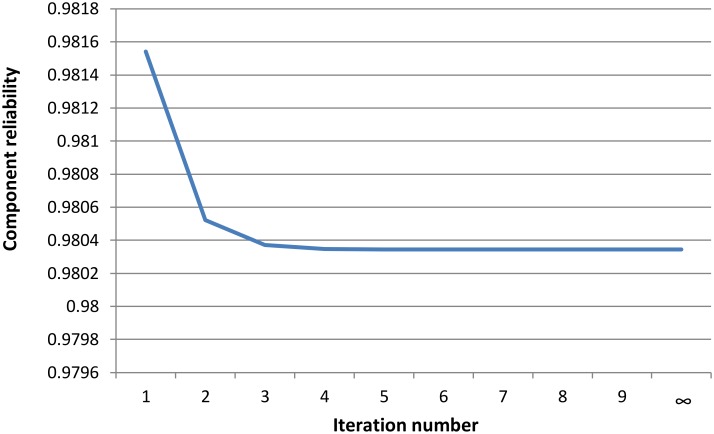
Impact of the iteration number on component reliability.

To compute the reliability of the component, we set the maximum iteration number of the algorithm to a large number (in order to reach the steady state probability). Based on the result shown in Table 2 and Fig 10, it can be seen that the component reliability gradually decreased as the iteration number increased. Thereafter, the component reliability became stable when the iteration number was>4. We can, therefore, report that the reliability at the beginning of the execution was 0.981540, while it decreased and became stable at a value of 0.980344. The iteration number indicates the number of the execution cycle. Based on this result, the reliability of the avoid-component was 0.980344 which refers to the steady state probability of not being in any failure state.

**Table 2:** Summary of the results of applying CR_Estimate algorithm.

Iteration number	Component reliability
0	0.981540
1	0.980521
2	0.980372
3	0.980348
4	0.980345
≥5	0.980344

In order to investigate the computational accuracy of the CR_Estimate Algorithm, we need to compare the computed reliability values with measured values of the component. In the early prediction known as the measured reliability value will not be available, due to the absence of runtime information. Furthermore, obtaining a measured value of individual component reliability is not an easy task. To obtain the reliability of individual component, we need to define all the failure modes caused by the targeted component. Note that the component usually used as part of other components, therefore it is difficult to determine which component is causing a specific failure independently. For instance, the avoid-component is part of *Subsumption* component of the wheelchair and some of operations’ execution of the avoid-component are complemented by an operation that belongs to the *Subsumption*. Thus, it is difficult to determine whether a specific failure was caused by the avoid-component or other operation in the *Subsumption*. In fact, as discussed in the introduction of this paper, the absence of the run time information which is used in the component reliability measurement is the main reason that leads to develop the CR_Estimate Algorithm and the related early prediction algorithms.

Therefore, in the investigation of computational accuracy of the algorithm we will try to measure the difference between the expected and computed reliability value of the component. The expected value can be elicited from information related to analogous components with similar operations and information obtained from a domain expert, such as the information shown in Table 1. The expected value is not a perfect to evaluate the individual component reliability, because it is information source that is inherently subjective and may be inaccurate, either due to the complexity of the component or to unexpected operational profiles of that component. However, it is used in this investigation just to give indicator whether the computed value is close to the expectation or not with the a help of other related works such as the work presented in the study [7].

If we have *N* operations in the component, and *F*_*i*_ is the expected failure probability of the i^th^ operation, the expected reliability of the component Ex (R_c_) can be elicited by the following equation:

$$Ex(R_{c})=1-\sum_{i=1}^{N}\left(F_{i}\right)$$ (5)

Based on Eq (5) and the operation failures of the avoid-component shown in Table 1, it is Ex(R_c_) = 0.973. Therefore, the difference between the computed value and the expected is 0.00734. By applying the same way for the algorithm presented in [7], based on the expected operation failures of the used component, the Ex(R_c_) = 0.910, while the computed was 0.9223, so the difference is 0.0123. Based on this simple comparison, our computed value seems more close to the expectation. The purpose of this investigation is just to check whether the accuracy of the computed value is logically acceptable or not. While the main comparison between this research and the related works including the work in [7] will be discussed in the comparison of the results (Section 4.6).

### Sensitivity Analyses

**Fig 11:**
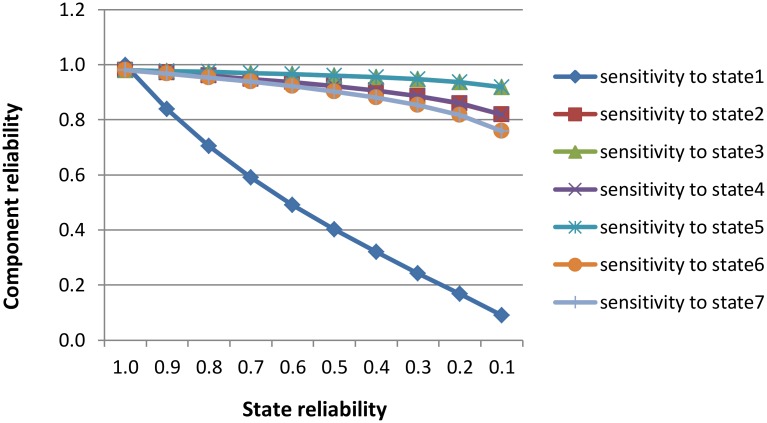
Analysis of sensitivity to states’ reliability.

Sensitivity analyses, which vary certain parameters of the behaviour model in order to perceive the effects on the prediction results, are an important and common way to gain more insights into the reliability characteristics of the component under study [2,16,39]. Using the CR_Estimate algorithm we investigated the variation in the reliability of the component as a function of the reliability of states. The graph in Fig 11 and the results in Table 3 show the impact of changing the state reliability with respect to the component reliability. In our analysis we maintained the reliability values of all the states assigned in our previous calculations and varied the reliability of one state at a time between 0 and 1.

**Table 3:** Tabular representation of Fig 11.

State reliability	R of comp (state1)	R of comp (state2)	R of comp (state3)	R of comp (state4)	R of comp (state5)	R of comp (state6)	R of comp (state7)
1	0.99825	0.980679	0.980241	0.980579	0.980300	0.980697	0.980439
0.9	0.838993	0.970535	0.977182	0.970453	0.977234	0.967507	0.967289
0.8	0.705382	0.959821	0.973813	0.959762	0.973859	0.953450	0.953276
0.7	0.590883	0.948332	0.970051	0.948300	0.970093	0.938237	0.938110
0.6	0.490741	0.935775	0.965774	0.935776	0.965813	0.921456	0.921383
0.5	0.401277	0.921710	0.960794	0.921754	0.960831	0.902486	0.902474
0.4	0.319424	0.905428	0.954798	0.905525	0.954836	0.880309	0.880370
0.3	0.242353	0.885642	0.947206	0.885807	0.947248	0.853070	0.853220
0.2	0.167019	0.859590	0.936732	0.859848	0.936784	0.816746	0.817014
0.1	0.089282	0.819032	0.919362	0.819433	0.919437	0.759150	0.759600

Table 3 is the tabular representation of the graph in Fig 11. The first column presents the state reliabilities. Each of the following columns shows the reliability of the component (R of comp) when the reliability of the state between brackets varied according to the values of the first column.

From Fig 11, it can be observed that the component reliability varied significantly with the variation in the reliability of *state1* which is related to the receipt of the signal from the IR sensor. As the reliability of this state decreased, the component reliability dramatically decreased. This is due to the fact that this state is at the heart of the avoid-component and therefore any faults in this state will easily propagate and affect the correct operation of the component. In addition, from our CPDG shown in Fig 9 above it can be observed that, as a minimum, *state1* will visit twice per each execution cycle. Furthermore, *state1* belongs to all CPDG paths. On the other hand, the reliability of the component doesn’t vary significantly with the variation in the reliability of *state3* and *state5*. This is due to the weak probability of visiting these; for example, the transition probability of *state3* only equals 0.0971. On the contrary, the component reliability is more sensitive to the reliability of *state2* and *state4*; this is due to their higher transition probability as compared to *state3*. Among the other states, *state6* and *state7* are similar to *state1*. Both *state6* and *state7* belong to more than one path in CPDG; thus, the component reliability is more sensitive to these states than in the case of all other states except *state1*.The analysis results demonstrate and identify the criticality of each state and the operation that led to it within the avoid-component clearly.

**Fig 12:**
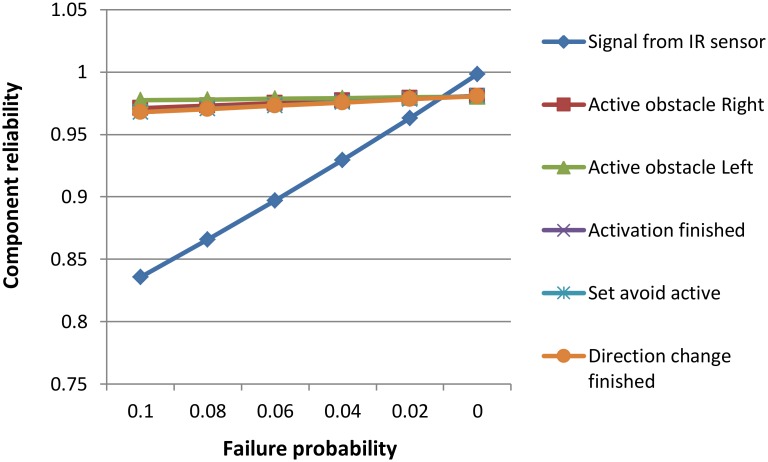
Analysis of sensitivity to operations’ failure probability.

Table 4 is the tabular representation of the graph in Fig 12. The first column represents operation failure probabilities. Each of the following columns shows the reliability of the component when failure probabilities of operation between brackets varies according to the values of the first column.

**Table 4:** Tabular representation of Fig 12.

Failure probability	R of comp (Signal from IR sensor)	R of comp (Activate obstacle Right)	R of comp (Activate obstacle Left)	R of comp (Activation finished)	R of comp (Set avoid active)	R of comp (Direction change finished)
0.1	0.835902	0.970920	0.977406	0.967953	0.967956	0.967743
0.08	0.865844	0.972942	0.978027	0.970594	0.970595	0.970374
0.06	0.896951	0.974942	0.978637	0.973201	0.973202	0.972974
0.04	0.929301	0.976923	0.979235	0.975778	0.975779	0.975542
0.02	0.962979	0.978884	0.979823	0.978326	0.978326	0.978082
0	0.998075	0.980828	0.980401	0.980846	0.980846	0.980594

Fig 12 illustrates the impact of varying the operations’ failure probabilities on component reliability. In each sensitivity run, the failure probabilities of a certain operation were varied between 0 and 0.1, while all other operations remained unchanged. We executed all runs under the obstacle avoidance scenario. As the results in the Fig 12 show, decreasing the failure probabilities generally grew the component reliability linearly. The operations *Activation finished*, *Set avoid active* with *Direction change finished* had a similar impact on component reliability, as did *Activate obstacle Right* with *Activate obstacle Left*. Component reliability was particularly sensitive to the *signal from IR sensor*, which plays the central role in avoiding an obstacle. The higher impact of this operation is due to the multiple invocations per each path traversal. Hence, it is most beneficial to focus on improving the reliability of the *signal from IR sensor* operation.

In conclusion, the derivation and identification of the component states, together with the operations that lead to them, in relation to reliability prediction for the avoid-component of the wheelchair system are indeed essential for identifying the critical states and operations in the component. This type of analysis result could provide valuable information that will enable a component’s architect to know the effectiveness of the states and the operations quantitatively. Moreover, sensitivity analyses on the wheelchair demonstrate that the proposed reliability technique is meaningful and useful from the perspective of making design decisions, as the reliability values obtained are able to aid the architect in evaluating design alternatives.

### Comparison of Results

This section investigates the improvement of the proposed technique as compared with the existing works. The investigation was based on a comparison with the related works as discussed above in Section 2. To fairly compare, we chose the three most similar techniques with the proposed technique, as shown in Table 5. The selected techniques were equally compared with the selected comparison criteria regarding early reliability prediction built on the behaviour model of the components. The comparison criteria were divided into two aspects, namely, the behaviour model and the computational model. The behaviour model aspect reflects the capability of capturing the component structure and behaviour. Moreover, whether the behaviour model that is used as architecture is fine-grained sequential model or not. On the other hand, the computational mechanism aspects concern the availability of the operational data and consideration of the loop entry and exit points relevant to the behaviour model in the reliability computation. The term fine-grained as mentioned in the introduction, according to [10] it refers to the use of a scenario language such as UML SD, MSC or LSC to describe the system scenarios, which have ability to reveal the dynamic behaviour of the system. Then identify an explicit mechanism for transforming these scenarios to state machine formalism such as LTS. Therefore, for any prediction technique if its architecture does not rely on such elements then it can be described as coarse-grained.

**Table 5:** Reliability prediction in design-time techniques.

Technique	Behaviour model		Computational model	
	Structure and behaviour	Fine-grained	Data availability	Loops
Rodrigues et al.[10]	√	√	×	×
Roshandel et al.[40]	(√)	×	√	×
Cheung et al. [25]	(√)	×	√	×
Proposed technique	√	√	√	√

The comparison results in Table 5 summarize the improvement of the proposed technique against the selected techniques. A √ mark in parenthesis means that the technique partially fulfilled the criteria. For the behaviour model aspect, the fine-grained criterion was fully supported by the proposed technique and by the technique proposed by Rodrigues et al. [10], but was neglected in the techniques proposed by Roshandel et al. [40] and Cheung et al. [25]. These two techniques represented the behaviour model of software as a provided state machine without showing how this model was derived from the requirements specification. In our technique and in the technique proposed by Rodrigues et al., the behaviour model is derived step by step in a precise way from the requirements specification using algorithm presented in our previous work [24] and discussed briefly in this paper. On the other hand, all the techniques rely on the structure and behaviour of the design specifications but this is partially included in Roshandel et al.’s and Cheung et al.’s works, where the scenario specifications as a primary source for identifying a dynamic behaviour of the system did not appear or were not explicitly used. As for the data availability criterion, most of the approaches provided a mechanism for generating the operational data except for the work by Rodrigues et al. which assumed the availability of such data. Only the proposed technique considered the loop entry and exit points in the computation using a stack-based algorithm; the other techniques used the DTMC to compute the reliability, which does not provide support for such factors.

From the comparison result in Table 5, in summary, the proposed technique is able to reveal the component’s structure and behaviour and provides fine-grained sequential models to be used as the base for reliability prediction. It depends on the requirements specification as input, which is a main source and can be available at the early design stage. The proposed technique takes into account the loop entry and exit points of the behaviour models in the reliability computation. Moreover, it considers the availability of operational data at the early design stage. The inclusion of these factors in the reliability computation can provide a realistic and meaningful evaluation of a component’s reliability. In this sense, the proposed technique shows a strong coupling between the requirements specification, design specifications and computation mechanism during the reliability prediction, which has been overlooked by most of the existing techniques.

## Conclusions and Future Work

In this paper, a technique for the early reliability prediction of software components is presented. The proposed technique is shown to have the potential to address the various challenges related to reliability prediction at the early design stage, such as capturing and modeling component behaviour based on the requirements specification. In the proposed technique, a state machine that represents a component’s behaviour is synthesized to reveal the component’s dynamic behaviour by describing all the possible interaction sequences of the component. The state machine is utilized as a base to generate the component-relevant operational data with the support of data gathered from similar function components, domain knowledge, the HMM, and the Baum–Welch algorithm. Moreover, the state machine is mainly used as a source for identifying the nodes and edges of a probabilistic dependency graph, called the CPDG. The generated operational data are used to identify the values of the CPDG parameters. Component reliability is computed through a tree transversal algorithm called the CR_Estimate which utilizes the CPDG as input.

The requirements specification of an ATM system was used to illustrate the applicability of the proposed technique. A case study for the control system of a robotic wheelchair system was used to evaluate the proposed technique. The evaluation results of applying CR_Estimate in the case study indicate that the proposed technique provides meaningful reliability prediction in the context of the early stages of software development. The results clearly identified the relationship between the execution cycles and the component reliability. In addition, the results identified the critical states of the component that would require intensive testing and validation. The comparison of our technique and the existing works showed that our proposed technique provides a realistic and meaningful evaluation of a component’s reliability, which leads to accurate prediction.

There are several open issues for future work. It is noted that the reliability prediction of software components may be applied to components and their behaviour in isolation, such as an off-the-shelf component, which is not part of any system. However, the prediction may be more meaningful when the component is considered in the context of a system. Therefore, we intend in future research to construct a system-level reliability approach that can utilize the results obtained through the proposed technique. Another plan is to enhance the accuracy of the operational profile that predicts the transition probabilities among the components’ states through incorporating other machine learning techniques such as the hierarchal hidden Markov model[41]. Furthermore, to broaden the applicability of the proposed technique to different application domains, our future work intends to apply it to a large number of components whose detailed requirements specifications are available. Another improvement related to the failure assumption in our work is that there is a possibility that the component might recover from the failure and successfully finish the task’s execution; such a consideration can be included in future work.
