# Correction: Technique for Early Reliability Prediction of Software Components Using Behaviour Models

**DOI:** 10.1371/journal.pone.0168722

**Published:** 2016-12-15

**Authors:** Awad Ali, Dayang N. A. Jawawi, Mohd Adham Isa, Muhammad Imran Babar

The affiliation for the second and third authors is incorrect. Dayang N. A. Jawawi and Mohd Adham Isa are not affiliated with #1 but #2 Department of Software Engineering, Faculty of Computing, Universiti Teknologi Malaysia, UTM, 81310 Skudai, Johor, Malaysia.
